# Tracing of Heavy Metals Embedded in Indoor Dust Particles from the Industrial City of Asaluyeh, South of Iran

**DOI:** 10.3390/ijerph19137905

**Published:** 2022-06-28

**Authors:** Mahsa Tashakor, Reza Dahmardeh Behrooz, Seyed Reza Asvad, Dimitris G. Kaskaoutis

**Affiliations:** 1 School of Geology, College of Science, University of Tehran, Tehran 14155-6455, Iran; mahsa.tashakor@ut.ac.ir; 2 Department of Environmental Sciences, Faculty of Natural Resources, University of Zabol, Zabol 98615-538, Iran; 3 Department of Environment, Faculty of Natural Resources and Marine Science, Tarbiat Modares University, Tehran 14115-111, Iran; reza.asvad@gmail.com; 4 Institute for Environmental Research and Sustainable Development, National Observatory of Athens, Palaia Penteli, 15236 Athens, Greece; 5 Environmental Chemical Processes Laboratory, Department of Chemistry, University of Crete, 70013 Heraklion, Greece

**Keywords:** potential toxic elements, household dust, petrochemicals, health risk, Asaluyeh, Persian Gulf

## Abstract

Assessment of indoor air quality is especially important, since people spend substantial amounts of time indoors, either at home or at work. This study analyzes concentrations of selected heavy metals in 40 indoor dust samples obtained from houses in the highly-industrialized Asaluyeh city, south Iran in spring and summer seasons (20 samples each). Furthermore, the health risk due to exposure to indoor air pollution is investigated for both children and adults, in a city with several oil refineries and petrochemical industries. The chemical analysis revealed that in both seasons the concentrations of heavy metals followed the order of Cr > Ni > Pb > As > Co > Cd. A significant difference was observed in the concentrations of potential toxic elements (PTEs) such as Cr, As and Ni, since the mean (±stdev) summer levels were at 60.2 ± 9.1 mg kg^−1^, 5.6 ± 2.7 mg kg^−1^ and 16.4 ± 1.9 mg kg^−1^, respectively, while the concentrations were significantly lower in spring (17.6 ± 9.7 mg kg^−1^, 3.0 ± 1.7 mg kg^−1^ and 13.5 ± 2.4 mg kg^−1^ for Cr, As and Ni, respectively). Although the hazard index (HI) values, which denote the possibility of non-carcinogenic risk due to exposure to household heavy metals, were generally low for both children and adults (HI < 1), the carcinogenic risks of arsenic and chromium were found to be above the safe limit of 1 × 10^−4^ for children through the ingestion pathway, indicating a high cancer risk due to household dust in Asaluyeh, especially in summer.

## 1. Introduction

Particulate matter (PM) is considered a major indicator of air quality, composed of various particles of different origin, size, chemical composition and toxicity [1]. In the urban agglomerations and industrialized areas, fossil-fuel combustion from vehicles and industries is the main source of PM pollution, which is escalated by emissions from residential heating and cooking, road dust resuspension and long-range transported aerosols [2,3,4]. Especially in urban areas largely affected by dust storms, PM concentrations can be highly increased during such episodes causing cardiovascular and respiratory diseases, asthma, lung cancer, etc. [5,6]. Among PM substances, various chemical compounds, such as polycyclic aromatic hydrocarbons (PAHs) and heavy metal(loid)s (e.g., Arsenic, As; Cadmium, Cd; Chromium, Cr; Lead, Pb; Nickel, Ni; Zinc, Zn), can adhere to dust surfaces, with deleterious effects on human health and ecosystems [7,8,9]. According to the International Agency for Research on Cancer (IARC), these substances are considered carcinogenic, and the World Health Organization (WHO) has defined annual limits for As (6 ng m^−^^3^), Cd (5 ng m^−^^3^), Ni (20 ng m^−^^3^) and Pb (0.5 ng m^−^^3^), in a way to control concentrations and protect human health.

People spend substantial amounts of time indoors, either in homes, offices or at schools, and for entertainment; this time was estimated at about 88% of the daily life for adults and 71–79% for children [10,11,12]. Therefore, indoor air quality is especially important for human health, while it depends on outdoor air pollution and human activities indoors such as cooking, cleaning and smoking [13,14,15]. Indoor air pollution is an issue concerning all people due to its deleterious effects on human health, thus attracting the interest of many researchers [16,17]. Similar to outdoor ambient conditions, household aerosols may be composed of a heterogeneous mixture of organic and inorganic compounds, including PAHs and heavy metals [18,19,20]. Furthermore, indoor dust particle may have considerable range in size, thus causing different effects on the human respiratory system, with the smaller and more toxic elements being more hazardous, as they can reach deep into the alveoli [21]. Other studies have shown that escalated outdoor PM concentrations from anthropogenic or natural sources (i.e., wildfires) negatively affect the indoor air quality, with indoor concentrations being even higher than outdoors [22]. Heavy metals and potentially toxic elements (PTEs) may originate from various sources in urban areas such as combustion emissions from vehicles, industrial zones, power plants, refineries and municipal waste disposal [23,24,25]. Heavy metals can enter the human organism via inhalation, corresponding to the airborne dust fraction, via ingestion due to contamination in food and via dermal contact [26,27].

Iran has been facing serious air pollution issues during the last decades due to rapid industrialization, urbanization and an increase in energy demands [28], which contribute highly to a background turbid atmosphere due to natural aerosols such as dust [29]. The Iranian economy is largely based on combustion of fossil fuels for energy production, exports of oil, natural gas and petrochemical products, which further deteriorate the urban environments [30,31]. The industrial sector is a major source of ambient air pollution, which may also highly contribute to indoor air quality, although this effect has not been well quantified [32]. Asaluyeh, located on the northern shore of the Persian Gulf, is one of the most highly industrialized cities in Iran, due to the operation of several oil refineries, petrochemical industries and the Pars Special Energy Economic Zone (PSEEZ). The gas and petrochemical industries release a large amount of pollutants such as volatile organic compounds (VOCs) and heavy metals [33,34]. Previous study in Asaluyeh examined the concentrations of PTEs and mineralogy of 43 street dust samples collected from industrial and urban areas in summer [35]. Statistical analyses revealed the main sources of trace elements to be the resuspended dust, traffic emissions and industrial sources. Another study analyzed the concentrations of heavy metals (Cu, Zn, Pb, Co, Cr, Fe, Ni and Mn) in dust deposited on surface and palm tree leaves at industrial, urban and non-urban areas in Bushehr and Asaluyeh, and assessed the contamination levels and possible sources [36], while a recent study evaluated the Hg concentrations of indoor dust samples in Asaluyeh against those in Ahvaz, southwest Iran and Zabol, southeast Iran [37].

As a measure of warning people for the deleterious effects of excessive indoor pollution, studies analyzing the dispersed pollutants and heavy metals indoors are very important, and also help in mitigation strategies for their reduction [17,38]. Although extensive analysis has been performed on PTEs in soil and airborne outdoor dust in Asaluyeh county [39,40,41], there are limited data related to heavy metal concentrations indoors. This study analyzes dust samples obtained indoors in the spring and summer seasons in Asaluyeh city, south Iran. The main objectives are (i) to determine the levels and distribution of selected heavy metal(oid)s of indoor dust in a highly-industrialized area, (ii) to compare the concentrations between different homes, areas and seasons, (iii) to estimate the health risk (carcinogenic and non-carcinogenic) posed by exposure to PTEs for children and adults via inhalation, ingestion and dermal contact pathways for the first time in Asaluyeh.

## 2. Material and Methods

### 2.1. Heavy Metal Concentrations

**Figure 1:**
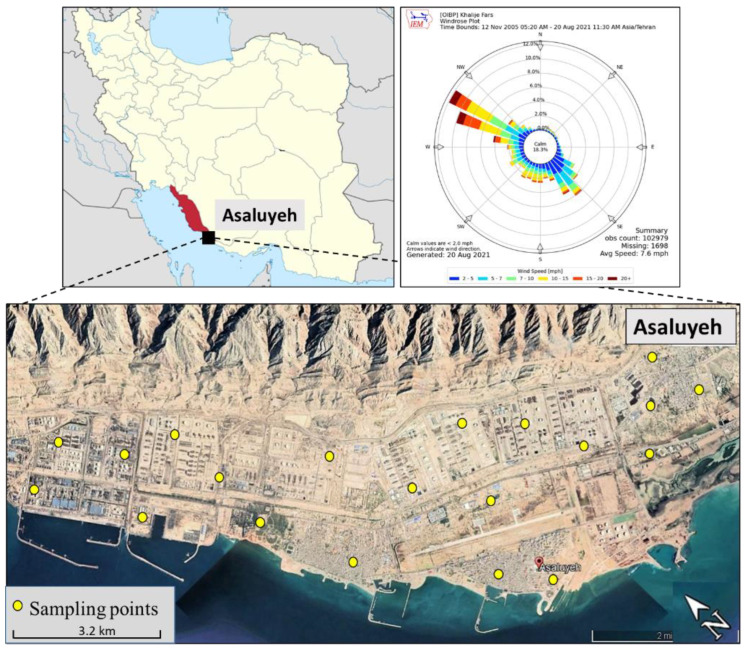
District distribution map of the Bushehr province in south Iran and the Asaluyeh county (**top left**) and the locations of the sampling points (houses) in Asaluyeh (**bottom**), along with wind rose diagram during the sampling days (**top right**).

Asaluyeh county, with an area of about 30,000 hectares, is situated in the Bushehr province, southwest Iran, between 27°14′–30°16′ N, and 50°6′–52°58′ E, limited by the Persian Gulf in the south and the Zagros Mountains in the north (Figure 1). The area experiences a hot and arid climate with annual temperatures ranging between 5 °C and 50 °C, relative humidity between 50% and 88% and average annual rainfall of 100 mm [36]. The prevailing wind direction is from northwest to southeast (Figure 1). The region has experienced remarkable industrial and economic activity due to the establishment of the Pars Special Energy Economic Zone (PSEEZ) in 1998 near Asaluyeh city, which covers a total area of 14,000 ha. This zone includes the world’s second-largest natural gas reserve and the largest oil and gas resources in Iran (16 gas processing plants and 15 petrochemical complexes), with a standard capacity for natural gas of 3.4 × 10^12^ m^3^, releasing a large amount of pollutants into the atmosphere [42,43,44]. The area also experiences a high frequency of dust storms, especially during summer [45]. The increased industrialization and fossil-fuel combustion escalated the levels of fine particulate matter, organic compounds and heavy metals in Asaluyeh, and studies examined the health risk of exposure to ambient conditions for local residents and people working in the industrial zones [32,44].

### 2.2. Indoor Sampling Measurements

In this study, we analyzed the concentrations of selected heavy metals via chemical analysis of 40 samples obtained indoors in spring and summer 2021 (20 samples of household dust in each season). These samples were collected at random district zones in Asaluyeh city, as shown in Figure 1. The surveyed residences were private dwelling units selected based on the willingness of landlords to participate in this study. Dust samples were collected from vacuum cleaner bags, using volunteers living in governmental houses in Asaluyeh city. In all the sampling days, the meteorological conditions were fair without strong winds or dust storms.

All the houses had an area of 90 m^2^, with two bedrooms and were 15 years old. All were built in the same way by the government for employees working in the South Pars zone. There were two to four people living in, and all the houses had only one air conditioner in the kitchen. Vacuum sampling of house dust was performed with a vacuum cleaner 9 times per season (three times per month). Sampling was performed (in three bags) and each bag contained 30 to 50 g of soil. All three bags were passed through a sieve after the sample was obtained. The soil that passed through the sieve was kept in plastic bags until the sample was transported to the laboratory and stored in the refrigerator.

Sampling of all parts of the house (bedrooms, living room, kitchen, etc.) was performed. The last cleaning time at each house was not accurately known, but it was assumed to occur within one week from the sampling time and likely contributes to the range of the heavy metal concentrations between the houses. About 30 to 50 g of soil was generally collected in each vacuum cleaner bag. All of these houses have the same architectural form and equipment in terms of construction. Dust samples were limited only to homes with non-smoking residents in order to eliminate the bias from smoke contamination in the concentrations of trace metals. Furthermore, during the sampling period in spring and summer, due to very hot conditions (35–40 °C), the domestic cooling via air conditions was at its maximum.

The content in the vacuum cleaner compartment was placed in plastic resealable bags, labeled and returned to the laboratory where they were kept in the fridge at 4 °C until chemical analysis. For chemical preparation and digestion of the samples, the following materials and tools were used: (i) 63-micron mesh sieve, (ii) PTFE Teflon tubes for digestion of samples, (iii) nitric acid 65%, (iv) hydrofluoric acid 40%, (v) perchloric acid 70% suprapure with very high purity, all provided by Merck, Germany. Polyethylene funnel, Whatman 42 filter paper and 25 mL bellows for filtering digested samples were also used. The dust samples were initially dried in an oven at 105 °C for 24 h and then sieved [17,46]. After drying the samples, approximately 0.25 g of soft sifted dust was weighed with a digital microbalance with an accuracy of 0.0001 g, and using polyethylene pipes for digestion and 7 mL of a mixture of HClO_4_-HF-HNO_3_ acids in 1:2:4 ratios, the samples were placed on a hot plate until the white vapor rose and the digestion process was complete. After steaming and drying the acid, the residue was dissolved in 2% nitric acid to a volume of 25 mL. Control samples were also prepared by repeating all digestion steps without the presence of samples [47]. Inductively Coupled Plasma Mass Spectrometry (ICP-MS; 7800 Series) was used to determine the concentrations of selected heavy metal(loid)s (As, Cd, Cr, Co, Ni and Pb). In order to increase the accuracy of the test and reduce the error rate, the quality control method was performed as follows: (i) all containers used in sampling, digestion and storage of samples were pre-soaked in dilute nitric acid (20%) for 24 h, then washed with distilled water and dried; (ii) during digestion, a blank sample was prepared for each group of samples and analyzed against other samples; the calibration curve was drawn using a blank sample and four standard samples and its accuracy was then confirmed using a control solution and a standard sample close to the middle concentration range (approximately once every 10 samples); (iii) the controls for all stages of the work should not differ by more than 2% from the initial curve; for every 15 samples, one sample was re-measured randomly and a standard sample was also selected and re-measured to ensure that the device was working properly. Soil standards (CRM: NIST 2710) for elements were also used to evaluate the accuracy of the measurement method and the recovery percentage. The recovery for the studied elements was 79% to 115%. Moreover, the concentration of elements in the control samples was between 0.004 and 1 μg mL^−^^1^, which was much lower than the amount of these contaminants in the dust samples. Replicate soil standards (CRM: NIST 2710) and dust sample measurements (repeat five times) had a relative standard deviation (RSD) < 5%.

The geochemical results were analyzed for descriptive statistics using Microsoft Excel and SPSS 23.0 software. The Shapiro–Wilk test was carried out to control the normality assumption of data and revealed non-normal distribution (*p* value ≤ 0.05) for all analyzed elements. Based on this result, the non-parametric Mann–Whitney U test was used to examine the difference in the concentrations of elements for indoor dust between spring and summer samples.

### 2.3. Health Risk Assessment

Similar to many studies examining the exposure to ambient outdoor dust, the health effects of PTEs in indoor dust were considered via the three major pathways of chemical ingestion, inhalation and dermal contact. The health risk assessment model introduced by the U.S. Environmental Protection Agency was applied to estimate the cancer and non-cancer risks associated with heavy metals in household dust samples in Asaluyeh. This model takes into account three major pathways of adult and children exposure to heavy metals: (i) intake by direct ingestion of dust particles, (ii) intake through mouth and nose breathing of resuspended particles (inhalation), (iii) intake via absorption from skin adhered dust particles (dermal contact). The average daily intakes of heavy metals (mg kg^−^^1^ BW d^−^^1^) received through the ingestion (ADI_ing_), inhalation (ADI_inh_) and dermal contact (ADI_drm_) pathways are calculated using the below formulas [3]:

(1) $${\mathrm{ADI}}_{\mathrm{ing}}=C_{s}\times \frac{\mathrm{IngR}\times \mathrm{EF}\times \mathrm{ED}}{\mathrm{BW}\times \mathrm{AT}}\times 10^{-6}$$

(2) $${\mathrm{ADI}}_{\mathrm{inh}}=C_{s}\times \frac{\mathrm{InhR}\times \mathrm{EF}\times \mathrm{ED}}{\mathrm{PEF}\times \mathrm{BW}\times \mathrm{AT}}$$

(3) $${\mathrm{ADI}}_{\mathrm{drm}}=C_{s}\times \frac{\mathrm{ESA}\times \mathrm{SAF}\times \mathrm{DAF}\times \mathrm{EF}\times \mathrm{ED}}{\mathrm{BW}\times \mathrm{AT}}\times 10^{-6}$$

The hazard quotient (HQ) represents the non-carcinogenic risk of a single element and was calculated by dividing the average daily intake (ADI) for each element and exposure pathway to a specific reference dose (Equation (4)). The overall non-carcinogenic hazards caused by multiple metals are accounted for by the hazard index (HI) [48] and calculated by summing the HQ values of the measured metals (Equation (5)).

(4) $${\mathrm{HQ}}_{e}=\frac{{\mathrm{ADI}}_{e}}{\mathrm{RfD}}$$

(5) $$\mathrm{HI}=\sum {\mathrm{HQ}}_{e}=\sum \frac{{\mathrm{ADI}}_{e}}{\mathrm{RfD}}$$

where RfD stands for the reference dose (mg/kg/day), established by the USEPA, as an estimation of the maximum allowable rate, and e represents the route of exposure (ingestion, inhalation, dermal contact). The occurrence of non-carcinogenic effects is more likely when HI value is greater than 1, whereas a value of HI lower than 1 indicates no significant risk.

The carcinogenic or cancer risk (CR) for each metal indicates the probability of developing cancer over a person’s lifetime due to exposure to that pollutant [24], and according to USEPA [49], the acceptable limits are in the range of 1 × 10^−6^ to 1 × 10^−4^. TCR is the sum of obtained carcinogenic risks from various exposure pathways. CR and TCR were calculated using the following equations (Equations (6) and (7)):

(6) $${\mathrm{CR}}_{e}={\mathrm{ADI}}_{e}\times {\mathrm{SF}}_{e}$$

(7) $$\mathrm{TCR}=\sum {\mathrm{CR}}_{e}$$

SF_e_ represents the carcinogenic slope factor from Regional Screening Levels for each pathway. The CR related to skin contact was calculated only for As, because the dermal slope factor has not been established for the rest of the examined elements. Ingestion and dermal contact slope factors for Cd and Ni were also not provided, while Co is not considered as a carcinogenic element, and therefore, no CR values were estimated. The CR values surpassing 1.00 × 10^−4^ are considered to be unacceptable with the potential for causing cancer during the individual’s lifetime, while the values below 1.00 × 10^−6^ are considered to be safe, without causing significant health effects. The values between 1.00 × 10^−6^ and 1.00 × 10^−4^ indicate tolerable risk [50].

## 3. Results

### 3.1. Heavy Metal Concentrations

**Figure 2:**
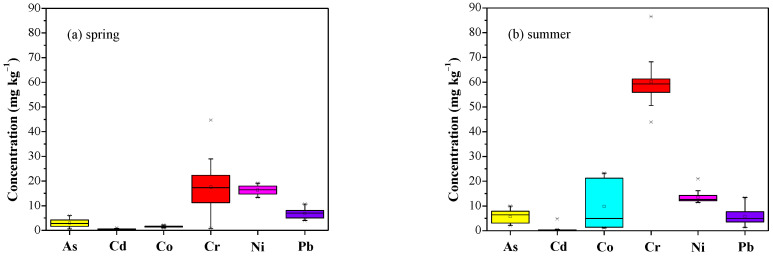
Box plots of the concentrations of heavy metals from indoor dust samples in Asaluyeh in spring (**a**) and summer (**b**). The bottom and top of the box are the first and third quartiles, respectively, while the mean is denoted by circle and the median by line within the boxes. Whiskers (the vertical lines) are the 1.5 interquartile ranges of the lower and upper quartiles.

The present study focuses on analyzing the concentrations of arsenic, cadmium, cobalt, chromium, nickel and lead, which are among the most dangerous types of trace element pollutants [51]. Metalloids such as arsenic (As) often fall into the category of heavy metals due to their similarity in chemical properties and environmental behavior [52]. Figure 2 shows the concentrations of the 6 examined heavy metals of household dust in Asaluyeh during spring and summer in a box–whisker chart view.

In both seasons, the highest concentrations occurred for Cr, followed by Ni, while in spring, concentrations of these elements were similar, although Cr exhibited a wider variability. In summer, Co concentrations were highly variable, while the mean was similar to that of Ni. For the collected household dust in summer, Cr exhibited the highest median concentration (59.3 mg kg^−^^1^), followed by Ni (12.6 mg kg^−^^1^), As (6.41 mg kg^−^^1^), Co (6.25 mg kg^−^^1^), Pb (4.91 mg kg^−^^1^) and Cd (0.24 mg kg^−^^1^). This pattern is slightly different in the spring collected samples, so that after Cr (17.3 mg kg^−^^1^) and Ni (16.5 mg kg^−^^1^), Pb exhibited higher concentrations (7 mg kg^−^^1^) than As (2.76 mg kg^−^^1^), Co (1.55 mg kg^−^^1^) and Cd (0.49 mg kg^−^^1^). Amongst the analyzed elements, Cr and Cd revealed the highest and the lowest concentrations, respectively, considering all available dust samples. Although Pb has been stopped as an additive in petrol and gasoline in Iran, Pb concentrations are likely due to the industrial sector, but also due to traffic emissions, as also shown in European urban sites [24,53].

**Figure 3:**
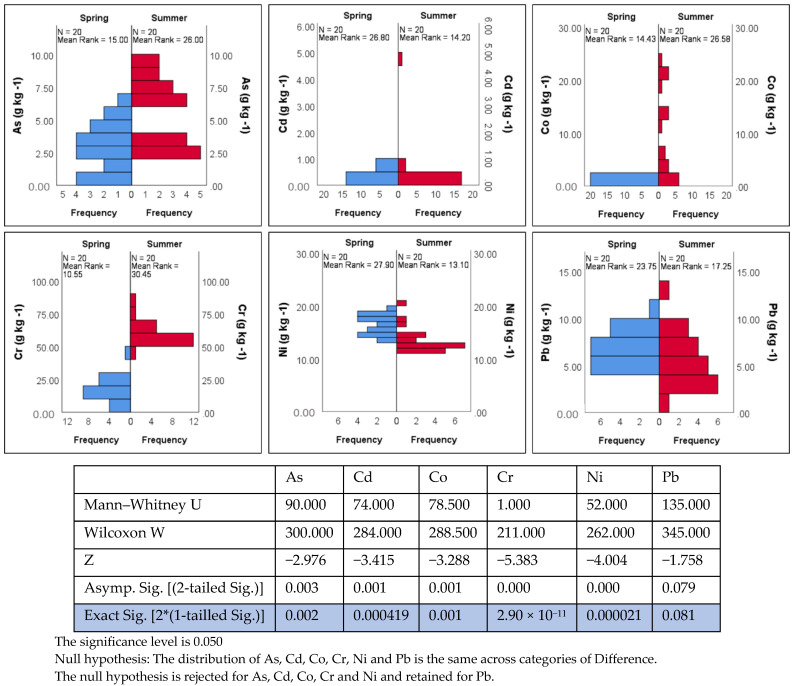
Histograms of the heavy metal concentrations and statistical results of the Mann–Whitney U test for examining the difference of the concentrations between spring and summer samples.

The influence of seasonal effect on the concentrations of trace elements was statistically evaluated by applying the Mann–Whitney U test. As can be seen in Figure 3, concentrations of As, Cd, Co, Cr and Ni significantly differ between spring and summer (*p* value < 0.01). This difference was not identified for Pb, which presented a *p* value larger than 0.05 (*p* value: 0.08). It should be noted that the concentration distributions for heavy metals were far from normality (Figure 3), so small changes in initial assumptions or data could lead to significant changes in *p* value. Especially for Cd, removing one outlier measurement from the summer dataset could significantly increase the *p* value.

Indoor air quality is highly affected by outdoor air conditions (infiltration of aerosols and pollutants) and indoor human activities, while the fine particles such as carbonaceous aerosols and heavy metals from urban/industrial emissions have higher infiltration rates than coarse desert-dust particles due to their smaller sizes [54,55]. The current results showed a general slight variation in PTE concentrations between the sampling homes in both seasons, except for Cr (in both seasons) and Co (in summer) where a larger variability was observed (Figure 2). In spring, the ratios between the highest and lowest concentrations were for Cr (~55), followed by As (~9), Cd (~4), Pb (~3), Co (~2) and Ni (~1). In summer, this ratio was Cd (~32), Co (~21), Pb (~11), As (~5), Cr and Ni (~2). In general, variation between the concentration of elements can be attributed to different sources of household dust particles, as well as different sources of metals introduced to each house [56]. Furthermore, the correlations between the PTEs were generally low, supporting the large heterogeneity in the indoor air pollution sources between the houses, while moderate correlations were found only between As and Cd (r = 0.31) and Cr (r = 0.51).

Descriptive statistics of heavy metal concentrations (in mg kg^−^^1^) for the analyzed indoor dust in Asaluyeh are shown in Table 1. Compared to the respective concentrations presented in studies of indoor dust samples from other regions in the world (Table 1), the obtained median values in this study, both from spring and summer samples, are either in the same range or significantly lower. Median concentrations of heavy metals in Asaluyeh were also below the limits defined for average global soils [57], except for As, which seems to be marginally higher in summer. Analysis of Asaluyeh surface sediments demonstrated a noticeable amount of arsenic released by diagenesis [58], while high concentrations of As in sediments from the Persian Gulf have also been mentioned in previous studies [59,60]. Previous work showed that Cr, Ni and Fe concentrations in street dust were lower in the industrial area of Asaluyeh compared to urban and non-urban areas in Bushehr [36]. Furthermore, the concentrations of Co, Cr and Ni can also be attributed to geogenic parameters, such as atmospheric inputs and the weathering of crustal materials [58]. The non-normal distribution of elements indicates the influence of multiple factors on the composition of indoor dust particles. Apart from the industrial and traffic emissions, increased dust activity and influence from Shamal dust storms increase the outside dust during summer, which may highly affect the concentrations of PTEs in indoor air samples.

The arid surroundings in Asaluyeh may highly affect the pollution levels, thus contributing to an already degraded urban environment due to refineries, petrochemical industries and large fossil-fuel combustion [32]. Furthermore, the high frequency of calcium carbonate in street dust in Asaluyeh can cause high arsenic immobilization [32]. This suggests that a large fraction of As, Cr, Fe and Ni is from local soil sources, while Pb is attributed to anthropogenic emissions. On the other hand, fine dust particles are more efficient in carrying heavy metals due to their higher specific surface area, and presence of clay minerals and organic matter in the soil [73]. A previous study in Asaluyeh showed that the mean concentrations of heavy metals in outdoor dust and soil samples decreased in the order of Fe (~11 g kg^−1^) > Mn (365 mg kg^−1^) > Zn (170 mg kg^−1^) > Ni (79 mg kg^−1^) > Pb (68.1 mg kg^−1^) > Cu (54 mg kg^−1^) > Cr (35.7 mg kg^−1^) > Co (11.7 mg kg^−1^) [36], which presented notable differences from the decreasing order of the heavy-metal concentrations of household dust, indicating large influence from human intervention and/or significant limitation of outdoor dust indoors. Similar results regarding the heavy metal concentrations from street dust samples in Asaluyeh were reported by Abbasi et al. [36]. This large difference between outdoor and indoor concentrations of heavy metals should be examined in more detail with new sampling strategies of concurrent outdoor and indoor measurements.

Although traffic is not considered as a major influential factor in Asaluyeh, proximity to the main roads, especially those driving to the PARS industrial zone, as well as to oil refineries and petrochemical plants may influence the variations in heavy-metal concentrations between the houses [74,75]. However, differences in human activities indoors is likely the most influential factor for the changes between concentrations of heavy metals in Asaluyeh. Compared to indoor heavy metal concentrations in other urban/industrialized areas around the world (Table 1), it is seen that the petrochemical industries in Asaluyeh have a small effect on indoor air quality. Higher heavy metal concentrations occur in cities larger than Asaluyeh (i.e., Istanbul, Cairo, Sydney, Toronto, Tokyo), where the traffic effect is especially high. Low traffic emissions in Asaluyeh is also a reason for the much lower PTEs concentrations in this city compared to other urban/industrial areas in Iran [32,36,37]. Continuous monitoring of indoor air pollution in Asaluyeh using gravimetric analysis of airborne PM_2.5_ samples, which is more health relevant to the citizens, is necessary and will allow apportionment of the sources affecting indoor air pollution.

Analysis of indoor air quality in two churches in Faisalabad, Pakistan revealed high concentrations of PM_2.5_ (69 ± 28 μg m^−3^), CO_2_ (1459 ± 714 ppm), NO_2_ (216 ± 37 ppm) and SO_2_ (125 ± 65 ppm), attributed to a crowded population and poor ventilation systems, as well as to high outdoor pollution [76]. Coal and biomass combustion was identified as the highest contributing source to measured polycyclic aromatic hydrocarbons (PAHs) in indoor dust in Kocaeli, Turkey [77]. On the other hand, indoor black carbon (BC) concentrations at households in rural areas across the Ganges valley, north India, were about 6–7 times higher when traditional cookstoves were used instead of liquefied petroleum gas for cooking, thus highlighting the importance of using new clean technologies for reducing indoor air pollution [78]. Recently, the lockdown intervention due to the COVID-19 pandemic significantly reduced the outdoor air pollution, but the longer stay and working at home resulted in an increase in indoor PM concentrations [79,80].

A previous study in Denver, Colorado during wildfire seasons revealed that indoor BC levels were about 2.4 times higher at homes that kept windows open for more than 12 h a day than homes with closed windows, while similar features were observed for PM_2.5_ and NO_x_ [22]. This indicates that different times of natural ventilation in homes by opening windows may highly differentiate the PM and PTE levels. Beyond open windows and doors, outdoor air pollutants can also enter indoors through mechanical air-ventilation systems and unintentional openings in the buildings. The construction rules for naturally ventilated buildings, the materials and the building air tightness are important factors determining the indoor air pollution levels [81,82]. Furthermore, the number of residents in each house may be an important determining factor for the levels of heavy metals indoors [17], although other studies have reported higher concentrations of Fe and Zn in houses with only 1–2 residents [72]. Another reason for the variation in heavy-metal concentrations between different houses may be the operation of air conditioning systems, which was found to be associated with increased levels of Cu, Cr, Cd and Mn in Istanbul [17]. On the other hand, recent studies have shown that paint walls are a significant source of heavy metals, since green is mostly associated with higher concentrations of Cu, purple with Zn and Pb and yellow with Cd, Cu, Pb and Zn [17,66,72]. Consequently, the time passed from the last wall painting and the quality of the paints used, may be a regulatory factor for the levels of heavy metals and the variability in concentrations between the houses.

### 3.2. Health Risk Assessment

#### 3.2.1. Average Daily Intake of HEAVY Metals from Indoor Dust

The average daily intakes of heavy metals through the ingestion (ADI_ing_), inhalation (ADI_inh_) and dermal contact (ADI_drm_) pathways in Asaluyeh, were calculated using the Equations (1)–(3). The results showed that ADI_ing_ was found to be the main pathway of heavy metal intake from indoor dust followed by skin contact and inhalation. The results showed that the average daily intake of all of the investigated heavy metals are higher in children than adults. The highest ADI_ing_ values were found for Cr (7.70 × 10^−4^ for summer and 2.24 × 10^−4^ for spring) and Ni (2.09 × 10^−4^ for spring and 1.73 × 10^−4^ for summer) in children (Table 2). Similarly, Cr and Ni showed the highest degree of average daily intake for ingestion in adults (5.33× 10^−5^ and 2.04 × 10^−5^, respectively). Regarding the minimum ADI_ing_, Cd revealed the lowest probability of intake by adults and children. The ADI_drm_ values were in the order of 10^−6^–10^−7^ for children and 10^−7^–10^−8^ for adults, while ADI_inh_ values were two orders of magnitudes lower (Table 2).

#### 3.2.2. Non-Carcinogenic Risk Assessment

**Figure 4:**
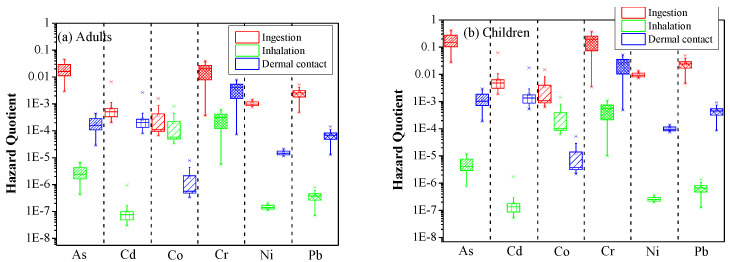
Hazard Quotient (HQ) values for non-carcinogenic health risk of selected heavy metals in household dust in Asaluyeh for adults (**a**) and children (**b**), and for the three pathways (ingestion, inhalation and dermal contact).

Regarding the non-carcinogenic risk, the estimates showed that the ingestion of dust particles was found to be the predominant pathway triggering non-carcinogenic risk in both adults and children, while inhalation was the lowest harmful pathway for all examined heavy metals except for Co (Figure 4).

The larger HQ values for children compared to adults revealed their higher vulnerability to heavy metals [27,83,84]. The highest HQ_ing_ was obtained for As in summer dust (4.25 × 10^−1^ for children and 4.55 × 10^−2^ for adults). However, As has still low potential to cause non-carcinogenic risk (0.1 < HQ < 1 for children and 0.01 < HQ < 0.1 for adults). The obtained HQ_ing_ values were also relatively high for Cr, revealing an average of 3.95 × 10^−2^ for adults and 3.69 × 10^−1^ for children. Regarding the HQ_inh_ and HQ_drm_, Cr was also recognized as the most hazardous element for indoor dust samples in Asaluyeh (Figure 4). The possible non-carcinogenic risk in case of long exposure through inhalation and dermal contact was the least for Cd and Co, respectively. The average HQ values for children showed to be one order of magnitude higher compared to adults.

**Figure 5:**
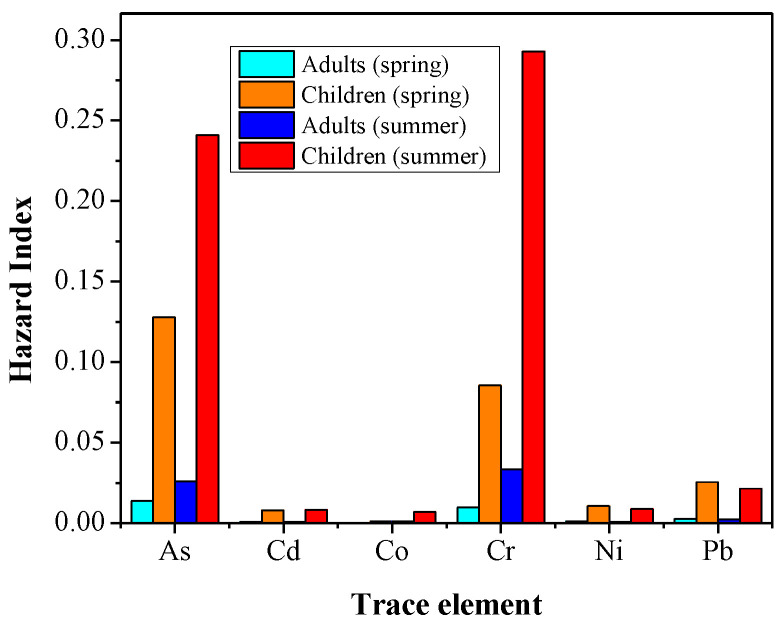
Hazard index (HI) values of selected heavy metals for non-carcinogenic health risk in Asaluyeh for adults and children.

The overall cumulative non-carcinogenic risks of heavy metals from household dust was found in the safe allowable limit of HI ≤ 1 for both sub-population groups (Figure 5). Due to lower pollution levels in spring, the exposure to PTEs reduced, and consequently, the risk to human health was lower via all the examined pathways. The median HI values decreased in the order of As > Cr > Pb > Ni > Cd > Co for the spring and Cr > As > Pb > Ni > Co > Cd for the summer samples. These orders are similar for adults and children though the trend of higher levels for children than adults was consistent for all of the elements, indicating that children are at higher risk by PTEs during their lifetime [85].

#### 3.2.3. Carcinogenic Risk Assessment

**Figure 6:**
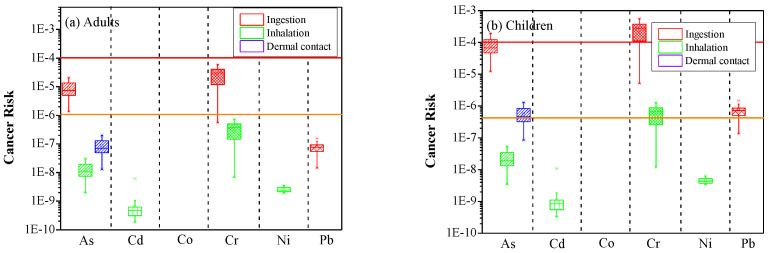
Cancer risk (CR) values of selected heavy metals in household dust in Asaluyeh for adults (**a**) and children (**b**), and for the three pathways (ingestion, inhalation and dermal contact). The orange and red lines denote the lower and upper safe limits, respectively.

Regarding the carcinogenic health risk (CR), ingestion of indoor dust particles was again estimated to be the foremost pathway threatening the health of Asaluyeh residents (Figure 6). The CR values for children were significantly higher than those for adults, while the main factors affecting this difference are the higher ingestion rate and the lower body weight of children [3]. The carcinogenic risks associated with oral intake of As and Cr were found to be within the tolerable thresholds for adults and at high risk (>10^−4^) for children. The cancer effect associated with the inhalation pathway was found to be insignificant for both adults and children (CR_inh_ < 10^–6^), except for Cr in children, while CR values above 10^−6^ were also found for Pb via ingestion and for As via dermal contact, but only for children (Figure 6).

**Figure 7:**
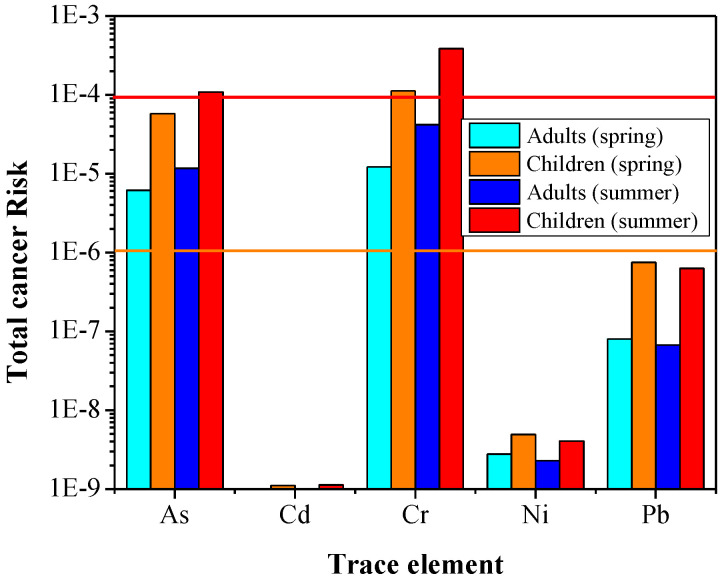
Total cancer risk values of selected heavy metals in Asaluyeh for adults and children. The orange and red lines denote the lower and upper safe limits, respectively.

The results of total cancer risk (TCR) suggested that As and Cr are the elements of major concern, with the potential for causing a lifetime risk of developing cancer (Figure 7). The TCR values for Cr ranged from 5.20 × 10^−6^ to 5.55 × 10^−4^ for children and 5.63 × 10^−7^ to 6.00 × 10^−5^ for adults, representing a moderate-to-high risk. Similar results were obtained for As, with the TCR values ranging from 1.26 × 10^−5^ to 1.92 × 10^−4^ for children and 1.35 × 10^−6^ to 2.07 × 10^−5^ for adults. Hence, the mean TCR values for children exceeded the safe threshold level of 10^−4^ for Cr in spring and summer, while the TCR for As was close to this level (10^−4^). For adults, TCR values for Cr and As were within the tolerable limits, while the carcinogenic risks posed by Cd, Ni and Pb were assessed to be very low (CR < 10^−6^) in both age groups. Men et al. [4] also reported higher CR for As, Cr and Ni in road dust in Beijing. Spring and summer dust samples followed the same trend of health risk; however, the TCR appeared to be higher in summer dust due to much higher concentrations of heavy metals (Figure 2).

## 4. Discussion

In residential areas located close to large industrial sectors, excessive accumulation of heavy metals in street and airborne dust can cause chronic respiratory diseases for local inhabitants through ingestion, inhalation and dermal contact routes [86,87]. Household dust plays a dual role in health exposure to PTEs, combining the transfer of outdoor dust and pollutants indoors [88,89]. The current results revealed that household dust in Asaluyeh industrial city can cause a cancer risk to the local population, mainly in children, through exposure to As and Cr, while ingestion was the riskiest pathway. Children are at the highest risk of exposure to PTEs, due to their physiological and behavioral characteristics (physical activity, playing in playgrounds, etc.) [90,91]. Furthermore, children are also more sensitive than adults to indoor air pollution, especially for the inhalation and ingestion pathways, as they breathe in a higher volume of polluted air relative to their body weight and have more frequent hand-to-mouth activities [92].

Heavy metals are of high concern for health-related issues due to their toxicity, bio-accumulation in human body and low degradation potential [93,94]. Cd and Pb were characteristic tracers of traffic emissions from engines and brake wear, while As, Cr and Ni are highly released from fuel combustion, among other sources [95,96]. Arsenic may cause deleterious effects on the human organism by negatively affecting the digestive, cardiovascular and DNA systems [97], and this is especially important in Asaluyeh due to increased CR values of As. Furthermore, Pb can negatively affect the kidneys, the soil pH and its absorption capacity, while high quantities of Pb can cause neurological disorders [8,98,99], although the indoor concentrations of Pb in Asaluyeh were not at such alarming levels. Exposure to excess Ni levels can be associated with increased carcinogenic risk and heart attack, as it can be bio-accumulated in the bones, liver, kidneys and aorta [100]. However, it should be noted that CR values for each element were mostly sensitive to the ingestion rate and skin adherence factor, which control the human health risk assessment [3].

Studies examining the health risk for indoor dust are few compared to those related to outdoor ambient or street dust. Similar to the current results, the HI values for eight PTEs in indoor dust in Istanbul were below the safe limit of 1 for both children and adults, indicating less potential health risk [17]. Ingestion was found to be the major pathway of exposure to indoor dust for non-cancer effects in homes and offices in Istanbul, followed by dermal contact and inhalation [17]. Our results showed highest CR for Cr in Asaluyeh, especially in summer, which was above the safe limit for children (Figure 5). The CR values of Cr for children in indoor dust in Istanbul was estimated at 3.7 × 10^−5^, 3.4 × 10^−10^ and 3.9 × 10^−5^ for ingestion, inhalation and dermal contact, respectively, which were within the acceptable limits of EPA. The respective CR values for adults were 2.7 × 10^−5^, 1.3 × 10^−9^ and 1.1 × 10^−4^, indicating significant risk for dermal contact [17]. On the other hand, a study in Vilnius, Lithuania showed that despite the much lower fine PM_1_ concentration indoors than outdoors (8.8 ± 2.7 μg m^−3^ against 43.2 ± 22.3 μg m^−3^) during forest fire events, the highly acidic indoor PM_1_ could have harmful health effects [101], thus highlighting the importance of aerosol PH in health-related studies. Furthermore, it should be noted that apart from the PTEs that are examined in this work, many other components of PM, such as VOCs, formaldehyde and carbon monoxide (CO), may negatively affect air quality and human health indoors [102,103].

Previous estimates of the health risk based on street dust samples in Asaluyeh revealed HI values below 1 and less health risk to residents, with ingestion being the dominant pathway for both children and adult [32], in accordance with the current results. It is worth mentioning that although the examined PTEs do not seem to cause non-carcinogenic risk for adults (HI < 1), the population of Asaluyeh is predicted to be exposed to morbidity and mortality risk due to long-term exposure to fine particulate matter generated by intense dust storms over the adjacent arid and semi-arid regions [104]. So, the combination of dust and heavy metals from oil refineries and petrochemical industries deteriorates air quality, leading to unhealthy conditions at least for sensitive population groups such as children and elderly. A recent study regarding the health effects of indoor mercury (Hg) in Asaluyeh showed moderate-to-low health risk and lower levels than those observed in the larger city of Ahvaz, southwest Iran [37]. A recent study in Zabol, southeast Iran [84] reported high non-carcinogenic and carcinogenic risks (HI > 1; CRs > 10^−4^) for children and adults due to exposure to outdoor dust. In developing countries of south Asia, despite awareness of the strong relationship between environmental degradation and human health, diseases and deaths related to atmospheric pollution have been increasing during the last decades [105,106].

Although widely used, the EPA model for health risk assessment is subjected to some biases, since the input variables (i.e., concentrations of heavy metals, ingestion rates, body weight, skin adherence factor) may not accurately represent the population groups at any time, and may vary among different people and even for the same person [3]. Evaluation of these uncertainties and sensitivity of each input variable to the health risk assessment were not considered in most studies, while Men et al. [3] provided such sensitivity analysis based on Monte Carlo simulations. They found that the cancer risk was more sensitive to heavy metal concentrations rather than other input variables, while the skin adherence factor and the ingestion rate were the most sensitive input variables for CR assessment.

## 5. Mitigation Strategies and Future Projections

In the hot environment of the Middle East, operation of cooling devices for avoiding opening windows under highly polluted atmospheres would be a good strategy to protect outdoor pollution from coming indoors, and this is recommended by public authorities during dust storms. This practice is also necessary during pollution events caused by accumulation of pollutants due to excess emissions and/or unfavorable meteorological conditions, such as calms, shallow boundary layer and temperature inversions. On the other hand, air filtering systems could be especially efficient in removing fine particulate from indoor air [101,107]. For new energy-conserving buildings in Asaluyeh, special treatment should be given to the ventilation options and in reducing exchanges between outdoor and indoor air in a way to limit health risk [108,109,110]. Therefore, improving air tightness of a building is an important pathway to limit the incoming of outdoor air pollutants, while mechanical ventilation with efficient air purification systems, such as HEPA, should be followed for highly polluted conditions [14,15,111].

This study provided first results concerning heavy metal concentrations and potential health risks due to exposure to indoor dust in Asaluyeh. Indoor and outdoor sampling will be continued in Asaluyeh city to assess possible changes in PTE concentrations and the effect of petrochemical industries and meteorology. More samples and chemical analysis will also allow for the determination of pollution sources in the city via statistical techniques, in order to evaluate the contribution of each source and to propose solutions. On the other hand, continuous sampling and assessment of the pollution sources and health risk will allow for warning strategies for the local population to be aware of the effects of short and long-term exposure to PTEs. Future research in Asaluyeh county in a way to prevent human health from exposure to petrochemicals and PTEs should emphasize (i) low-cost sensors that can provide real-time measurements of PM concentrations [112], (ii) analysis of PAHs and other organic compounds such as formaldehyde [113], both in outdoor and indoor air, (iii) dissemination of the pollution levels to local population via apps in smart phones or in public walls. Furthermore, more studies are recommended to be undertaken on spatial distribution patterns and source identification of PTEs in Asaluyeh and other highly-industrialized areas along the Persian Gulf due to highly turbid atmospheres from the combination of traffic and industrial emissions and dust storms. Continuous recordings of hospital admissions and statistics for morbidity and mortality in Asaluyeh county will further help in associating pollution levels and potential health risk assessment with hospitalization and morbidity rates.

## 6. Conclusions

This study analyzed the concentrations of selected heavy metal(oid)s (As, Cd, Co, Cr, Ni and Pb) of household dust in the industrial city of Asaluyeh in south Iran. The key objectives were to assess the indoor air quality and health risk for children and adults due to exposure to heavy metals via three pathways, i.e., ingestion, inhalation and dermal contact. The dust samples were collected at houses in Asaluyeh during spring and summer (20 samples in each season) and analyzed for heavy metals through Inductively Coupled Plasma Mass Spectrometry (ICP-MS).

The mean concentrations of heavy metals in the analyzed household dust samples followed the order of Cr > Ni > Pb > As > Co > Cd in both seasons, while significant differences were observed from spring to summer, with a large increase in mean Cr concentrations (60.3 mg kg^−1^ against 17.6 mg kg^−1^ in spring), as well as in As (5.62 mg kg^−1^ vs. 2.98 mg kg^−1^) and Co (9.85 vs. 1.54 mg kg^−1^).

Estimates of the health risk for the local residents due to exposure to household dust revealed generally low and acceptable levels for non-carcinogenic effects, since HI values were below 1 for both children and adults. The highest health risk was through the ingestion pathway, followed by dermal contact and inhalation, while As and Cr were the most hazardous elements for human health. Regarding the cancer risk (CR), the ingestion pathway for children presented values above the safe threshold of 10^−4^, while for adults the CR for ingestion was in the order of 10^−5^. Cr and As exhibited the highest CR values, while As through dermal contact, Cr through inhalation and Pb through ingestion also presented tolerable CR values for children, but lower than 10^−6^ for adults. Although in most of the cases the HI and CR values were within the acceptable limits of EPA (HI < 1; CR: < 10^−6^), the bioaccumulation of heavy metals to the tissues and other organs of the human body can cause chronic deleterious effects that could not be overlooked, especially in highly industrialized areas with several oil refineries and petrochemical industries.

## Figures and Tables

**Table 1:** Total concentrations of heavy metals (mg kg^−1^, dry weight) from indoor dust samples in Asaluyeh and other urban areas, and for average global soils.

Locations		As	Cd	Co	Cr	Ni	Pb	Reference
Asaluyeh (Spring)	Min	0.65	0.25	0.99	0.81	13.4	4.05	This study
	Max	6.02	0.98	2.17	44.7	19.1	10.6	
	Mean	2.98	0.49	1.54	17.6	16.4	6.87	
	Med	2.76	0.49	1.55	17.3	16.5	7.00	
	Std. Dev	1.66	0.18	0.29	9.66	1.85	1.80	
	Skew	0.19	1.16	0.44	0.91	−0.13	0.32	
	Kurt	−0.98	1.92	0.84	2.21	−1.44	-0.43	
Asaluyeh (Summer)	Min	2.10	0.15	1.12	43.9	11.4	1.24	This study
	Max	9.96	4.80	23.22	86.5	20.9	13.4	
	Mean	5.62	0.50	9.83	60.2	13.5	5.79	
	Med	6.41	0.24	6.25	59.3	12.6	4.91	
	Std. Dev	2.68	1.02	8.17	9.09	2.37	2.89	
	Skew	0.06	4.38	0.48	1.20	1.98	0.92	
	Kurt	−1.63	19.4	−1.37	3.01	4.27	1.02	
Ahvaz, Iran		n.a	0.25–0.65	5.8–11.8	10–26	5–20	39.6–124	Neisi, et al. [61]
Neyshabur, Iran		n.a	0.5–12.9	1.3–21.4	28.1–190	24.7–162	13.7–5345	Naimabadi, et al. [62]
Giza and Cairo, Egypt		n.a	2.23	n.a	68.1	39.2	222	Hassan [63]
Istanbul, Turkey		n.a	0.8	5	54.9	263	28.1	Kurt-Karakus [17]
Ilorin, North central Nigeria		0.08	0.12	3.35	1.92	1.35	5.55	Abdulraheem, et al. [64]
Chengdu, China		n.a	2.37	n.a	82.7	52.6	123	Cheng, et al. [65]
Kwun Tong, China		n.a	39	n.a	n.a	n.a	308	Tong and Lam [66]
Selangor, Malaysia		n.a	190	n.a	n.a	830	850	Latif, et al. [18]
Warsaw, Poland		n.a	n.a	n.a	90	30	124	Lisiewicz, et al. [67]
United Kingdom		n.a	1.3	n.a	n.a.	56.5	181	Turner and Simmonds [68]
Toronto, Canada		n.a	1.7	n.a	42	23	36	Al Hejami, et al. [56]
Ottawa, Canada		4.1	4.3	8.8	69	52	222	Rasmussen, et al. [69]
Tokyo and Hiroshima, Japan		n.a	1.02	n.a	67.8	59.6	57.9	Yoshinaga, et al. [70]
Christchurch, New Zealand		n.a	5.2	n.a	n.a	n.a	724	Kim and Fergusson [71]
Sydney, Australia		n.a	1.6	n.a	65	15	76	Chattopadhyay, et al. [72]
Average World Soils		6.83	0.41		60	18	27	Kabata-Pendias [57]

**Table 2:** Reference dose values of heavy metals for the ingestion, inhalation and dermal contact pathways used in this study.

	Population	ADI_ing_		ADI_inh_		ADD_der_	
		Spring	Summer	Spring	Summer	Spring	Summer
As	Adult	4.08 × 10^−6^	7.70 × 10^−6^	6.00 × 10^−10^	1.13 × 10^−9^	1.63 × 10^−8^	3.07 × 10^−8^
	Children	3.81 × 10^−5^	7.18 × 10^−5^	1.06 × 10^−9^	2.01 × 10^−9^	1.07 × 10^−7^	2.01 × 10^−7^
Cd	Adult	6.75 × 10^−7^	6.89 × 10^−7^	9.93 × 10^−11^	1.01 × 10^−10^	2.69 × 10^−9^	2.75 × 10^−9^
	Children	6.30 × 10^−6^	6.43 × 10^−6^	1.76 × 10^−10^	1.80 × 10^−10^	1.76 × 10^−8^	1.80 × 10^−8^
Co	Adult	2.11 × 10^−6^	1.35 × 10^−5^	3.10 × 10^−10^	1.98 × 10^−9^	8.40 × 10^−9^	5.37 × 10^−8^
	Children	1.97 × 10^−5^	1.26 × 10^−4^	5.49 × 10^−10^	3.51 × 10^−9^	5.50 × 10^−8^	3.52 × 10^−7^
Cr	Adult	2.40 × 10^−5^	8.25 × 10^−5^	3.54 × 10^−9^	1.21 × 10^−8^	9.59 × 10^−8^	3.29 × 10^−7^
	Children	2.24 × 10^−4^	7.70 × 10^−4^	6.27 × 10^−9^	2.15 × 10^−8^	6.28 × 10^−7^	2.16 × 10^−6^
Ni	Adult	2.24 × 10^−5^	1.86 × 10^−5^	3.30 × 10^−9^	2.73 × 10^−9^	8.95 × 10^−8^	7.40 × 10^−8^
	Children	2.09 × 10^−4^	1.73 × 10^−4^	5.85 × 10^−9^	4.84 × 10^−9^	5.86 × 10^−7^	4.85 × 10^−7^
Pb	Adult	9.42 × 10^−6^	7.93 × 10^−6^	1.38 × 10^−9^	1.17 × 10^−9^	3.76 × 10^−8^	3.16 × 10^−8^
	Children	8.79 × 10^−5^	7.40 × 10^−5^	2.46 × 10^−9^	2.07 × 10^−9^	2.46 × 10^−7^	2.07 × 10^−7^

## Data Availability

The data can be available upon request.
